# TLR4 Agonist Monophosphoryl Lipid A Alleviated Radiation-Induced Intestinal Injury

**DOI:** 10.1155/2019/2121095

**Published:** 2019-06-03

**Authors:** Jiaming Guo, Zhe Liu, Danfeng Zhang, Yuanyuan Chen, Hongran Qin, Tingting Liu, Cong Liu, Jianguo Cui, Bailong Li, Yanyong Yang, Jianming Cai, Fu Gao

**Affiliations:** ^1^Department of Radiation Medicine, College of Naval Medicine, Naval Medical University, 800, Xiangyin Road, Shanghai 200433, China; ^2^Department of Neurosurgery, Changzheng Hospital, Navy Medical University (Second Military Medical University), Shanghai 200003, China; ^3^Department of Nuclear Radiation, Shanghai Pulmonary Hospital, Tongji University, 507, Zhengmin Road, Shanghai 200433, China

## Abstract

The small intestine is one of the most sensitive organs to irradiation injury, and the development of high effective radioprotectants especially with low toxicity for intestinal radiation sickness is urgently needed. Monophosphoryl lipid A (MPLA) was found to be radioprotective in our previous study, while its effect against the intestinal radiation injury remained unknown. In the present study, we firstly determined the intestinal apoptosis after irradiation injury according to the TUNEL assay. Subsequently, we adopted the immunofluorescence technique to assess the expression levels of different biomarkers including Ki67, *γ*-H2AX, and defensin 1 in vivo. Additionally, the inflammatory cytokines were detected by RT-PCR. Our data indicated that MPLA could protect the intestine from ionizing radiation (IR) damage through activating TLR4 signal pathway and regulating the inflammatory cytokines. This research shed new light on the protective effect of the novel TLR4 agonist MPLA against intestine detriment induced by IR.

## 1. Introduction

The small intestine is highly sensitive to ionizing radiation (IR), and intestinal damage is one of the major side effects induced by radiotherapy. Irradiation of the intestine could result in diarrhea and other symptoms, which limits the further application of radiotherapy. On the other hand, the radiation injury on the intestine is even lethal when acute radiation syndrome, especially the gastrointestinal type, occurs. Till now, the only FDA-approved radioprotective drug used clinically is WR2721 (Amifostine, 2-(3-aminopropylamino) ethylsulphanyl phosphonic acid; marketed as Ethyol® by MedImmune, Gaithersburg, MD), which is, unfortunately, not free of toxicity as well. Therefore, novel safe and effective radioprotectants are urgently needed to mitigate the damages of IR on the small intestine.

Toll-like receptors (TLRs) are a class of pathogen-associated molecular patterns (PAMPs), and several members of TLRs were proved to be radioprotective. Burdelya et al. reported that TLR5 agonist CBLB502 effectively mitigated gastrointestinal and haematopoietic radiation injury in both mice and monkeys [1]. Moreover, we have demonstrated that TLR4 played a critical role in basal radioresistance by activating the NF-*κ*B signaling pathway [2]. And lipopolysaccharide (LPS), a classic TLR4 ligand, effectively protected intestinal injury through cyclooxygenase-2 (Cox-2) with the induction of prostaglandin E2 (PGE2) synthesis [3]. However, these TLR ligands are still away from clinical application due to their severe toxicity. Also, the exact mechanism of their radioprotective effects is still unclear.

Monophosphoryl lipid A (MPLA), an effective TLR4 agonist with low toxicity, is now clinically used as vaccine adjuvants, such as Fendrix (hepatitis B) and Cervarix (human papillomavirus-16 and human papillomavirus-18) [4]. MPLA is produced by hydrolysis of native diphosphoryl lipid A, which is the component of LPS recognized by TLR4. But compared to LPS, MPLA is 10,000 times less toxic, and over 300,000 human tests in vaccine have proved its safety [5]. And several studies have proved the strong TLR4-activating capacity of MPLA [6, 7]. In this study, we aimed to investigate the radioprotective effect of MPLA on intestinal damage induced by IR. And we found that MPLA effectively alleviated intestinal histological injuries, inhibited cell apoptosis, and restored immune dysregulation in a TLR4-dependent manner.

## 2. Materials and Methods

### 2.1. Mice and Treatment

All of our experimental procedures and protocols were well approved by the Second Military Medical University of China in accordance with the Guide for Care and Use of Laboratory Animals published by the US NIH (publication no. 96-01). C57BL/6 mice of wild-type (6-8 weeks old, China Academy of Science, Shanghai, China) and TLR4-deficient mice (TLR4-/-, Model Animal Research Center of Nanjing University) from the same background were selected for the animal experiments. Mice were housed in daily-changed individual cages at 25 ± 1°C with free access of food and water. Each mouse was administrated with either MPLA (1 *μ*g/mouse in 0.1 ml physiological saline) or physiological saline (0.1 ml/mouse) in accordance with the corresponding groups by gastrogavage 12 h before IR. On the third day after IR, mice were euthanized and small intestines were isolated for the subsequent experiments.

### 2.2. Irradiation

The ^60^Co *γ*-rays of Radiation Center of the Second Military Medical University was adopted for the irradiation treatment as described previously [8]. Mice were confined in the plastic chamber and received 7 Gy whole body *γ*-rays at a rate of 1 Gy/min. Then, mice were put back into the cages and housed for another 3 days.

### 2.3. Preparation of Paraffin Section for Histopathological Studies

Histopathological studies were conducted here as described elsewhere [9]. All the assays of this kind adopted in the current paper were based on the preparation of paraffin section of small intestines. Generally, mice were sacrificed and intestines were isolated, washed with PBS, and fixed with 4% paraformaldehyde shaking at 4°C overnight. The next day samples were rinsed with PBS twice and preserved in 50% ethanol at 4°C. After collecting one batch of desired samples, we embedded them in paraffin and cut them into thin sections of 4 *μ*m, which were readily for immunostaining of different molecules including Ki67, TUNEL, *γ*-H2AX, and defensin 1.

### 2.4. TUNEL Assay

The TUNEL assay was applied according to the manufacture's instruction with a little modification (Roche, Welwyn Garden, UK). Generally, the paraffin sections were subjected to deparaffinization with xylene and rehydration through a series of decreasing concentrations of ethanol. After rupturing the membrane, all the sections were incubated with the solution of TdT and dUPT (2 : 29) at 37°C for 2 h. Then, 3% H_2_O_2_ was applied to block the endogenous peroxidase. Subsequently, the slides went through several steps including decoloration, DAB coloration, counterstaining of nuclei, dehydration, and finally mounting and were ready for assessment by a fluorescence microscope in a double-blind method (Olympus BX60).

### 2.5. Immunofluorescence of Ki67, *γ*-H2AX, and Defensin 1

Immunofluorescence was carried out as previously described. Generally, the prepared sections underwent several steps including antigen retrieval, permeabilization, blocking, immunostaining, and counter staining sequentially. Finally, all the slides were mounted and analyzed via a fluorescence microscope (Olympus BX60). The primary antibodies of Ki67 (ab16667), *γ*-H2AX (ab2893), and defensin 1 (ab63982) were bought from Abcam company.

### 2.6. Real-Time PCR Assay

Total RNA was extracted from small intestines by using RNeasy kits (Qiagen, Valencia, CA) according to the manufacture's instruction. Subsequently, cDNA was prepared with SuperScript II kits (Invitrogen). The abundance of mRNAs was determined by real-time PCR as described elsewhere using primer pairs listed below:

1. TNF*α*: forward, catgcgtccagctgactaaa; reverse, tccccttcatcttcctcctt
2. IL-6: forward, gctgacctctggacgcttac; reverse, gggagatgcttttgttccaa
3. IL-1*β*: forward, ggcactttccagacttgctc; reverse, ctgttgagggattgggaaga

### 2.7. Statistical Analysis

All the data were presented as mean ± standard deviation (S.D.) from at least 3 independent experiments. Moreover, a two-tailed unpaired Student's *t*-test was adopted to determine the statistical significance between groups. Statistical analyses were performed via SPSS software (version 21.0). GraphPad Prism 5 Software (GraphPad software Inc., California, USA) was utilized to complete the graphs. *P* < 0.05 was considered to be significantly different.

## 3. Results

### 3.1. MPLA Inhibited IR-Induced Apoptosis via a TLR4-Dependent Way

**Figure 1:**
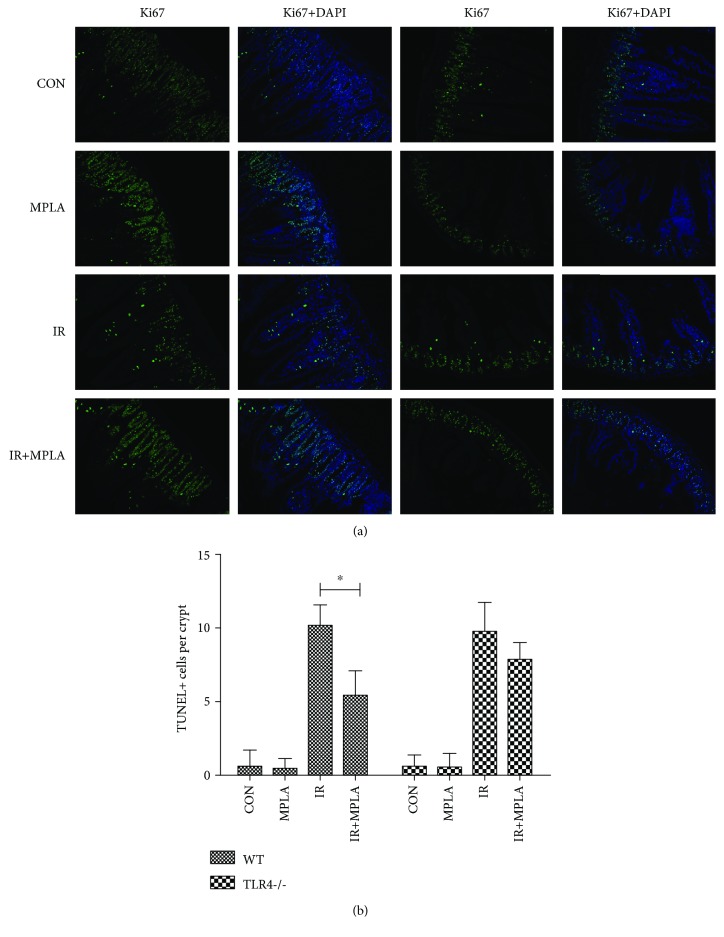
TUNEL analysis of intestinal apoptosis induced by IR exposure (7 Gy). (a) Representative pictures of the TUNEL assay indicate that MPLA reduces intestinal apoptosis signals of irradiated mice only in WT mice. (b) Quantitative comparisons of TUNEL-positive cell number per crypt among different groups. ^∗^*P* < 0.05. Data are expressed as mean ± S.D.

The first set of questions is aimed at determining whether MPLA could exert radioprotective effects against intestinal injuries, for which we adopted TUNEL assay to examine the histological intestinal cell apoptosis among different groups. The data showed that IR greatly increased TUNEL signal in mouse intestinal epithelium, which was significantly reduced by MPLA in WT mice but not in TLR4-/- mice (Figures 1(a) and 1(b)), indicating that MPLA alleviated IR-induced intestinal cell apoptosis through a TLR4-dependent manner.

### 3.2. MPLA Promoted the Proliferation of Intestinal Epithelial Cells (Ki67)

**Figure 2:**
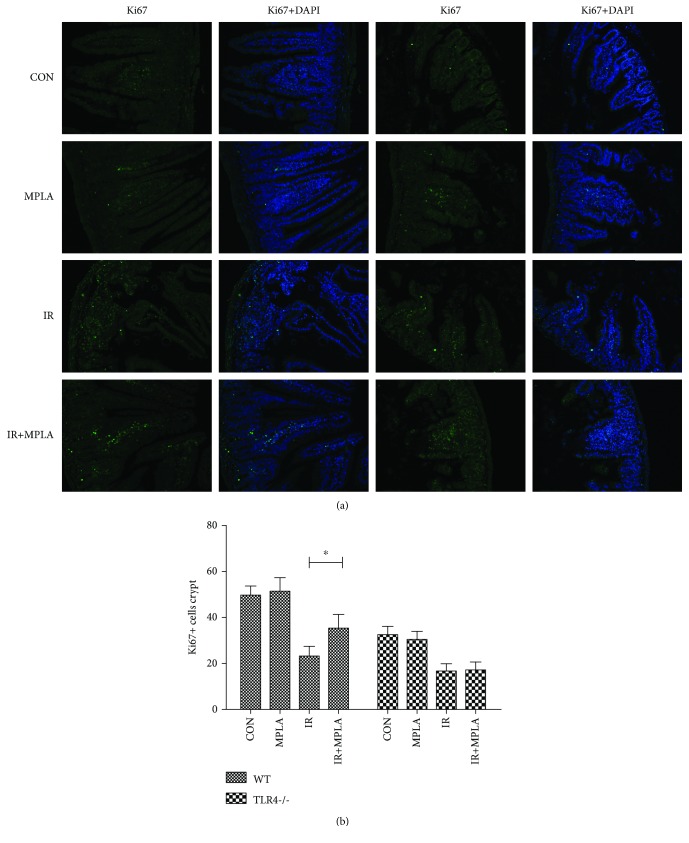
Immunostaining of Ki67 and its quantitative analysis. (a) Descriptive immunofluorescence pictures showed an increase of Ki67 dots caused by MPLA group compared to IR group. But this phenomenon did not appear in the TLR4-/- group. (b) Statistical analysis of the Ki67-positive cells in each crypt between different groups presents the similar trend as (a) does. ^∗^*P* < 0.05. Data are expressed as mean ± S.D.

Rapid proliferation is one of the most important characteristics of the intestine and plays a vital role in maintaining normal physiological function of the alimentary tract. Thus, we applied Ki67 immunostaining to assess the proliferative ability of mouse intestinal epithelium in various groups. Our results, as shown in Figure 2, indicated that IR decreased the Ki67 signal remarkably, which was reversed well by MPLA for the WT mice but not for the TLR4-/- ones. Therefore, these data suggested that MPLA could significantly restored the IR-damaged proliferation of intestinal epithelium in a TLR4-dependent manner.

### 3.3. MPLA Reduced the DNA Damage Caused by Irradiation

**Figure 3:**
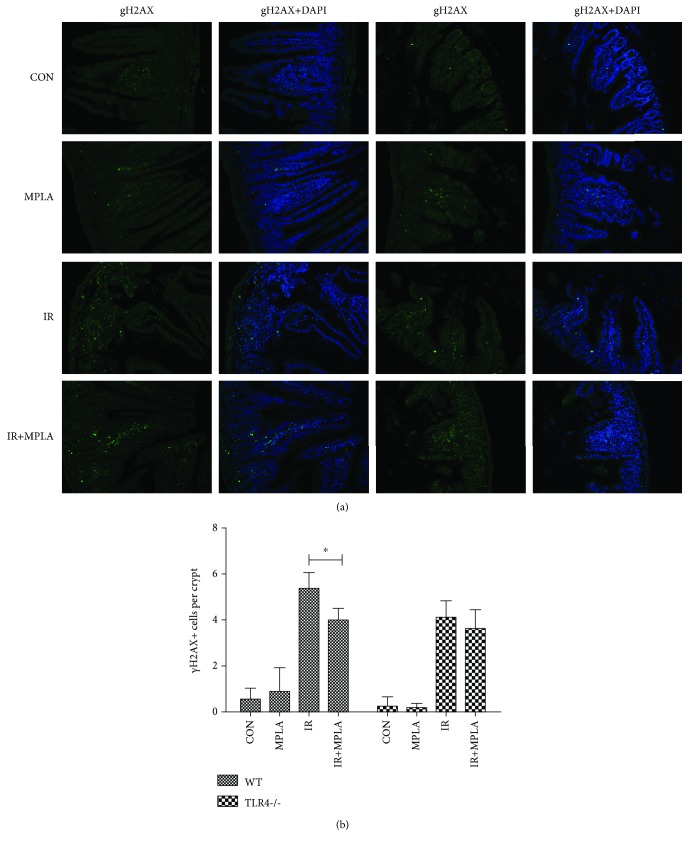
Immunofluorescence photos were taken under a fluorescence microscope for the detection of *γ*-H2AX formation. (a) Representative data randomly chosen from the corresponding group. (b) Calculation and comparison of *γ*-H2AX-positive cells in each crypt. It was clear that MPLA greatly inhibited the IR-elevated *γ*-H2AX formation in the WT mice intestine from both figures. ^∗^*P* < 0.05. Data are expressed as mean ± S.D.

We then investigated whether MPLA could influence DNA damage induced by IR using *γ*-H2AX assay. According to the descriptive pictures, much more *γ*-H2AX fluorescence from the IR-treated mice were detected compared with that from the control group, suggesting that IR caused severe DNA damages along the intestinal epithelium (Figure 3(a)). Moreover, the elevated DNA injuries were greatly alleviated when MPLA was administrated to the WT mice. Similarly, MPLA failed to work in TLR4-/- mice (Figure 3(a)). After counting and comparing the average number of *γ*-H2AX-positive cells in each crypt, we got the quantitative data with the same trend (Figure 3(b)).

### 3.4. MPLA Exhibited Little Influence on Defensin 1 Cells with or without TLR4

**Figure 4:**
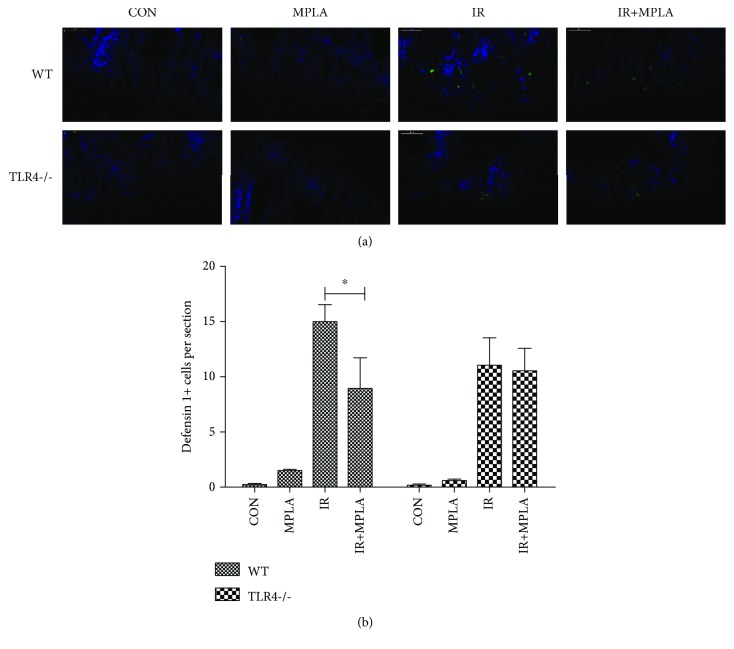
Detection of defensin 1 using immunofluorescence staining. (a) Representative pictures showed that the signals of defensin 1 were much increased by IR. However, no evident differences were found between IR and MPLA+IR group. (b) The quantitative data were well in keeping with the representative results above. ^∗^*P* < 0.05. Data are expressed as mean ± S.D.

In order to explore the influence of MPLA on the immune function of the intestine, we examined the defensin 1 expression via immunofluorescence technique. However, whether for WT mice or for TLR4-/- mice, no significant differences were found between IR group and IR+MPLA group (Figure 4), suggesting that some mechanisms other than adjusting defensin 1 may exist for MPLA to affect intestinal immune function.

### 3.5. MPLA Suppressed the Increase of Intestinal Inflammatory Cytokines Induced by IR

**Figure 5:**
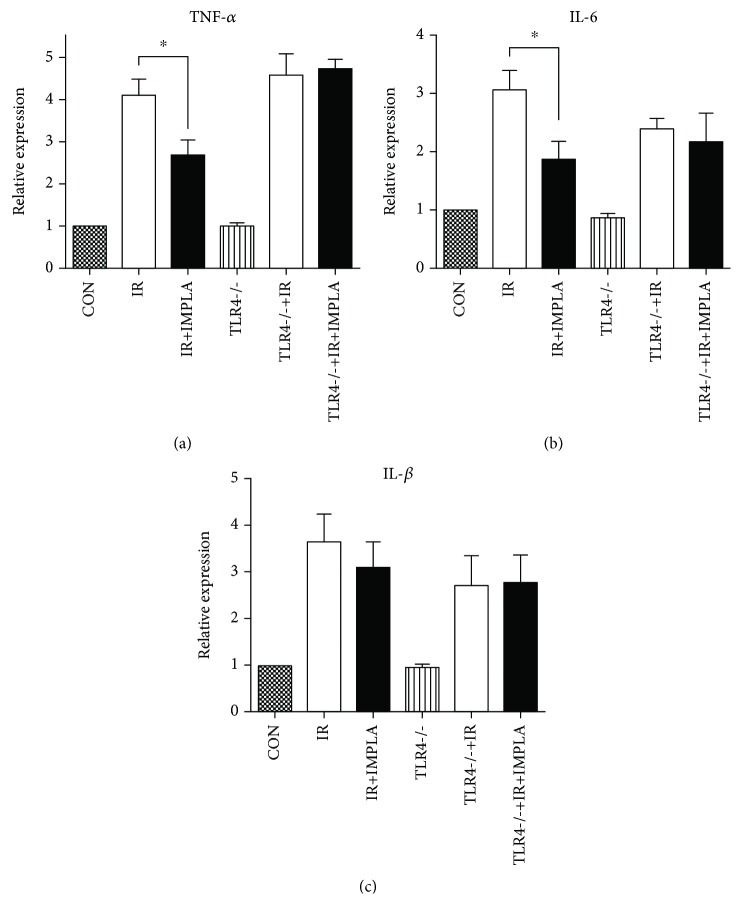
Determination of inflammatory cytokines including TNF-*α*, IL-6, and IL-1*β* via RT-PCR technique. (a) and (b) indicate a significant correction effect upon the IR-induced elevation of both TNF-*α* and IL-6 in a TLR4-dependent manner. But MPLA exerted no influence on IL-1*β* expression according to (c). ^∗^*P* < 0.05. Data are expressed as mean ± S.D.

Further, we continue to seek the underlying mechanism for MPLA-regulating immune function of the intestine using RT-PCR technique. As is known to us, IR frequently ignites burst of inflammatory cytokines among various tissue and subsequently exerts harmful impacts. From the data in Figure 5, we can see that IR elevated the levels of TNF-*α*, IL-6, and IL-1*β* greatly, whether in WT mice or in TLR4-/- mice. In addition, MPLA managed to repress the surged levels of both TNF-*α* and IL-6 for WT mice, but not for the TLR4-/- mice. What is more, MPLA failed to influence the IL-1*β* expression even for the WT mice (Figure 5). Our results indicated both anti-inflammatory and immunomodulatory effects of MPLA against IR-induced intestinal injuries.

## 4. Discussion

As a radiosensitive organ, the small intestine is prone to the radiation damage [10–12]. It is indicated that toll-like receptors (TLRs) are associated with the basal resistance to IR [2]. The agonists or ligands of TLRs are potentially radioprotective, while most of them are limited to animal experiment because of the severe toxicity. Our previous data have suggested that MPLA can significantly reduce the radiation damages to the spleen, the bone marrow, the intestine, and the testis, literally with little toxicity [2]. In the present study, we further examined the radioprotective value of MPLA on damage of the small intestine induced by IR. We found that MPLA could significantly inhibit the apoptosis of intestinal cells, promote cell proliferation, reduce DNA damage, and suppress inflammatory cytokines via TLR4 pathway, but it has little effect on the secretion of defensins.

IR can cause structural and functional changes in the small intestine, inducing cellular apoptosis and intestinal immune barrier damage [13–16]. Upon the radiotherapy of cancer, we aim to induce apoptosis of tumor cells, while the intestinal crypt epithelial cells are sensitive to IR and their apoptosis result in mucosal damage [14]. IR gradually kills the epithelial stem cells in crypt, and then, epithelial cells migrate into the intestinal lumen. As a result, crypts cannot be filled with the epithelial cells and subsequently involute. In such situation, the normal barrier of the small intestine is damaged and the lamina propria is exposed to luminal microorganisms [17], inducing acute inflammatory responses related to immune cell infiltration. These immune cells can release mediators and enzymes and lead to the damage of epithelial cells and degradation of extracellular matrix [16, 17]. Damage of mucosa and submucosal tissue is initiated by the destruction of critical cellular biomolecules attributable to the generation of radicals from radiolysis of water [17–19]. These free radicals may interact further with oxygen to extend the radiological effect. Consequently, severe damage to important cellular components is induced by IR, such as proteins, lipids, and DNA [20]. It is generally accepted that DNA damage is key to the cell apoptosis and it can be characterized by the formation of micronuclei and aberrations of chromosomal [18]. We found that MPLA can significantly reduce IR-induced DNA damage in intestinal tissues. IR can increase intestinal cell apoptosis and decrease cell proliferation, which can be significantly suppressed by MPLA. Moreover, we detected that these effects do not exist in TLR4-/- mice, indicating that MPLA plays the radioprotective role via TLR4-related pathway.

Paneth cells are radioresistant cells located at the bottom of crypts close to multipotent stem cells [21–23]. They play an important part in the natural defense of the intestine [22, 24, 25]. After being stimulated by bacteria or bacterial products, Paneth cells can release lysozymes and antibacterial peptides into intestinal cavity and then exert proinflammatory effects [22, 26]. Defensins are among the released peptides, which can inhibit bacterial activity, excite antigen-presenting cells rapidly, then upset the proinflammatory responses, and activate the immune responses [24–27]. It is suggested that Paneth cells secrete antibacterial molecules and tumor necrosis factor-*α* (TNF-*α*) upon oxidative stress to induce apoptosis and damage of epithelial cells [28]. IR could contribute to the extensive morphological changes in the small intestine such as shedding of villi, rupture of goblet cells, and submucosal contractions [29]. Moreover, previous studies report the upregulation of autophagy in Paneth cells, which can promote inflammatory remodeling in the intestine in response to IR [30, 31]. In the present study, MPLA attenuated the Paneth cell damage induced by IR, but had no effect on the secretion of defensins, indicating possible neutral effect of MPLA on the protective value of Paneth cells.

Furthermore, we investigated the effect of MPLA on cytokines after IR. The secretion of proinflammatory cytokines such as TNF-*α* frequently occurred besides morphological changes of the small intestine after IR injury [32]. In the study of serum cytokines after radiation, we found that MPLA could significantly reduce levels of IL-6 and TNF-*α*, suggesting the anti-inflammatory role of MPLA in IR. It is generally known that the gut microbiota plays a vital role in the maintenance of intestinal integrity and homoeostasis. And the major alterations in gut microbiota composition after radiation were reduced diversity of the Firmicutes and Bacteroidetes and increase in Proteobacteria. There are many studies regarding the role of changed gut microbiota in the pathogenesis of radiation enteritis while only a few of them tried to seek the underlying mechanism [33]. Recently, Shiran et al. reported that radiation-induced dysbiotic microbiota transmitted inflammatory susceptibility and rendered germ-free recipient mice more susceptible to radiation damage, which unveiled part of the explanations and suggested the microbiota manipulation as potential clinical interventions to ameliorate or prevent radiation-induced intestinal tissue damage. In our study, MPLA, the chemically modified production from native diphosphoryl lipid A that originates from the gram-negative bacteria, performed excellently in mobilizing the immunological responses while restraining the excessive inflammatory cytokines and exerted radioprotection against radiation injury in intestinal mucosa. Therefore, our data are consistent with Shiran et al.'s in that MPLA ameliorates the inflammatory microenvironment to resist the mucosa inflammatory susceptibility transmitted by radiation-induced dysbiotic microbiota. Thus, besides microbiota manipulation, application of new adjuvants such as MPLA may serve as another powerful tool to act against the complications of abdominal radiotherapy.

MPLA is a TLR4 agonist with low toxicity [2, 34–36]. Generally, TLRs play their roles via MyD88 or TRIF. In our study, the radioprotective value of MPLA was obliterated in TLR4 knockout mice, indicating that MPLA exert protective effect in TLR4-dependent manner. A study reported that MPLA caused gene expression primarily through TRIF [37]. Another study showed that the immune response after MPLA treatment was significantly affected by the deficiency of MyD88 or TLR4 [36], suggesting the possible role of MyD88 in MPLA signal. In our previous in vitro study, we found that MPLA reduced the cell apoptosis mainly via MyD88 other than the TRIF branch [2]. However, further studies with respect to the specific intestinal cell types or population should be conducted to clarify this obscure.

A variety of TLR ligands or agonists have been reported to be protective against IR. We found in our previous publication that MPLA could effectively defend cultured cells and radiosensitive tissues against IR via TLR4 signal pathway [2]. These results threw light upon the clinical application of TLR ligands. Efficacy and safety should be simultaneously considered in the clinical trials of radioprotectors. As a TLR4 agonist with low toxicity, MPLA demonstrates radioprotective effects against intestinal damage induced by IR [5, 6]. Nevertheless, since the present study was only conducted on mice, generalization to humans should be made with caution. So further researches need to be performed in the future.

In conclusion, our findings indicated that MPLA significantly protected the intestinal cells from IR via TLR4 signal pathway, which could also suppress the release of inflammatory cytokines in the small intestine caused by IR. This study presented a novel radioprotector of intestinal radiation injury with low toxicity and a great potential for clinical applications.

## Data Availability

The data used to support the findings of this study are available from the corresponding author upon request.
